# Integrating Structured Peer Support Pathways for Patients Undergoing Cellular Therapies: Insights from a Participatory Study

**DOI:** 10.3390/curroncol32100586

**Published:** 2025-10-21

**Authors:** Karine Bilodeau, Pegah Torabi, Ludovic Tamaro, Sandie Oberoi, Deborah Pascale, Kelley Kilpatrick, David Ogez, Marie-Pascale Pomey, Katia Dumont, Catherine Paquette-Gascon, Israel Fortin, Isabelle Fleury, Imran Ahmad

**Affiliations:** 1 Faculty of Nursing, University of Montréal, Montreal, QC H3T 1A8, Canada; catherine.paquette-gascon@umontreal.ca; 2 Maisonneuve-Rosemont Hospital Research Centre, Montreal, QC H1T 2M4, Canada; pegah.torabi@umontreal.ca (P.T.); katia.dumont@umontreal.ca (K.D.); imran.ahmad.med@ssss.gouv.qc.ca (I.A.); 3 Centre Intégré Universitaire de Santé et de Services Sociaux de l’Est-de-l’Île-de-Montréal, Montreal, QC H1T 2M4, Canada; soberoi.hmr@ssss.gouv.qc.ca (S.O.); deborah.pascale.cemtl@ssss.gouv.qc.ca (D.P.); israel.fortin.med@ssss.gouv.qc.ca (I.F.); isabelle.fleury.med@ssss.gouv.qc.ca (I.F.); 4 Susan E. French Chair in Nursing Research and Innovative Practice, Ingram School of Nursing, McGill University, Montreal, QC H3A 2M7, Canada; kelley.kilpatrick@mcgill.ca; 5 Department of Anesthesiology and Pain Medicine, Université de Montréal, Montreal, QC H3C 3J7, Canada; david.ogez@umontreal.ca; 6 Centre de Recherche du CHUM, Montreal, QC H2X 0A9, Canada; marie-pascale.pomey@umontreal.ca; 7 Cellular Therapy & Transplantation Program, Maisonneuve-Rosemont Hospital, University of Montréal, Montreal, QC H3T 1J4, Canada

**Keywords:** CAR-T, hematological cancer, hematopoietic cell transplantation, participatory action research, patient engagement, peer support, unmet need, tailored support, continuity of care, implementation

## Abstract

**Simple Summary:**

Individuals diagnosed with blood cancers treated with cellular therapies experience intense stress and frequently report insufficient personalized emotional support. To respond to these needs, our team co-designed a structured support program in collaboration with patients, healthcare professionals, and subject matter experts. At the heart of this program are Accompanying Patients (APs, *patients accompagnateurs* in French)-trained peers who have lived through cancer and are positioned to provide guidance and support throughout critical stages of care. In discussions with 16 stakeholders, we examined patient support needs, the contribution of APs, and the key conditions required for program implementation. Thematic analysis revealed that APs can play a crucial role in addressing emotional, social, and practical challenges, enhancing patients’ understanding of treatment, and fostering continuity of care. Participants also highlighted the need for well-defined roles, comprehensive training, and access to appropriate tools. This initiative marks a pioneering effort in Canada to explore the integration of peer support within the context of cellular therapies.

**Abstract:**

Individuals diagnosed with hematological cancer often face an emotionally demanding journey, frequently reporting high levels of distress and unmet needs, including a lack of personalized and emotional support. Our research team co-developed structured support provided by trained Accompanying patients (APs) during key stages of the care pathway for individuals with hematological cancers treated with cellular therapies. This paper presents findings on the anticipated needs that APs can address, their role within the care team, and the key facilitators, challenges, and strategies needed to implement structured peer support. Using a participatory action research approach, three working sessions were conducted with 16 key stakeholders to co-develop the structured support pathways. Data from group discussions (*n* = 3) underwent thematic analysis to identify essential components for adapting the support offered by APs, as well as the barriers and facilitators to its implementation. Findings show that APs can help address a wide range of practical, emotional, social, and psychological needs while enhancing patient understanding and continuity of care. Participants emphasized the importance of clear role definitions, adequate training, and supportive tools to ensure the safe and effective involvement of APs. Organizational and communication challenges were also identified, along with strategies to promote long-term sustainability. This initiative represents the first structured peer support program tailored to cellular therapies in Canada and offers a promising model to improve patient experience, foster relational continuity, and support cancer survivors across the care continuum.

## 1. Introduction

Hematological cancers affect nearly 7% of the global population diagnosed with cancer [1]. Over recent years, the 5-year survival rate for these cancers has seen a remarkable rise, increasing by 20–50 percentage points [2]. This significant improvement in survival can be attributed to groundbreaking advancements in medical care and treatments, particularly the innovations in cellular therapy (such as chimeric antigen receptor T-cell therapy (CAR-T)) and Hematopoietic Cell Transplantation (HCT) [3,4]. However, unlike individuals diagnosed with solid tumors, who often follow a more predictable disease course, those with hematological malignancies face a highly uncertain trajectory due to factors such as poor prognosis, high recurrence rates, and the complexity of treatments [5,6,7].

Individuals diagnosed by hematological cancer often experience greater treatment-related toxicities, psychological distress, and a lower quality of life [7,8]. They also report more severe symptoms, higher hospitalization rates, and more frequent admissions to intensive care units [9]. It is documented that this population faces numerous unmet needs at the end of care pathways, particularly related to emotions, fatigue, and concerns about cancer recurrence [10,11]. These individuals experience different and unique needs at various stages of the disease, as the episodic nature of cancer care and treatment evolves [7,12]. However, it is difficult for them to share their concerns including emotional, physical, and other personal needs, due to the psychological impact of cancer diagnosis and treatment [13]. Many hide their emotions and needs from their families in effort to minimize distress and other psychological burdens on their loved ones [14,15]. They may struggle to meet their own needs due to the shock of diagnosis, the distress of hospitalization, and feeling lost both in the healthcare system and in their daily lives [16]. Moreover, healthcare support in hematology-oncology often falls short in recognizing the unique experiences of these individuals and providing timely, personalized support [17].

For cancer survivors, the lack of tailored support, combined with their frustration over unmet needs, diminishes their sense of control, adding an additional burden to their cancer journey [13].

Nevertheless, accompanying patients (APs)—also known as peer supporters—who have personally experienced cancer, can help individuals diagnosed with cancer express their feelings more openly, strengthen their resilience, and alleviate common challenges such as depression and anxiety [18,19]. They can act as patient advisors, drawing on their firsthand knowledge of managing the disease, navigating healthcare services, and engaging with healthcare professionals (HCPs) [20]. By sharing their experiences, they provide a valuable source of support for patients and HCPs, which not only strengthens their own well-being and confidence but also improves the health of patients currently undergoing cancer treatment [20]. They can contribute across the entire cancer care continuum, promoting patient engagement at every stage, assisting with treatment adherence, and providing guidance to both patients and their families [21,22]. Therefore, the support of APs, which allows for authentic experience sharing, has become an indispensable resource in cancer care [23].

Several challenges have hindered the widespread integration of APs in cancer care, including the diverse information needs of patients with different cancer types and the lack of structured guidance and training [24,25]. In this context, the Government of Quebec (Canada) has made patient partnership a strategic priority in its health and social services system, supported by concrete policy actions and legislative reforms aimed at restructuring healthcare institutions to foster its integration into clinical, organizational, and strategic practices [26]. Building on this commitment, Province of Quebec has also emerged as a pioneer through the implementation of peer support programs such as PAROLE-Onco, which has developed and documented effective strategies for integrating APs into clinical oncology teams, particularly for specific cancer populations like individuals with breast cancer [27]. However, this type of support has not yet been implemented or evaluated in hematological oncology, particularly in the context of cellular therapies such as HCT or CAR-T cells.

To fully engage APs in supporting cancer survivors, it is essential to develop a structured AP support system along with standardized guidance and information [18,24,28]. Therefore, well-structured AP support and tailored guidance are needed to better understand and address the unmet needs of individuals affected by cancer, particularly those who have received CAR-T therapy or allogeneic HCT, as these treatments often lead to severe complications, prolonged hospitalizations and greater unmet needs.

To address these challenges, our team has developed support guides to outlining two structured support pathways provided by trained APs during key stages of the care pathway (e.g., start of treatments/hospitalization, end of treatment). These pathways were co-designed in collaboration with key stakeholders—including APs, HCPs, and managers—to tailor the components of structured support tailored to the care pathways of CAR-T and allogeneic HCT. This paper reports on the anticipated needs to be addressed by an AP, the role of APs, and the key facilitators, challenges, and strategies for the implementation of structured support offered by an AP.

## 2. Materials and Methods

**Figure 1:**
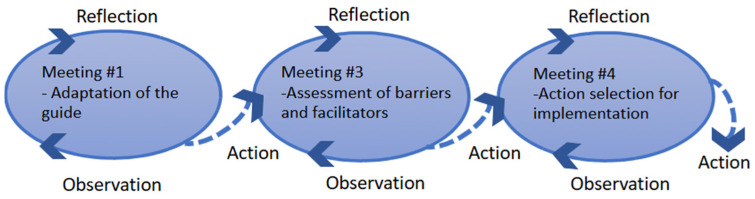
Reflective cycles during meetings for each working group.

This research is based on the participatory action research approach [29]. This research approach engages both researchers and knowledge users in a reflective process that generates useful and accurate knowledge, aligned with the users’ realities and needs, while also identifying practical solutions to common challenges [29,30]. This approach fosters synergy through partnerships with knowledge users, including individuals living beyond hematological cancer, HCPs, and managers, which enabled us to conduct the study in a way that was both culturally appropriate for the target population and logistically feasible [31]. A distinctive feature of the participatory research approach is its use of reflexive cycles during data collection, which involve observation (describing/defining), reflection (analyzing/interpreting), and action (identifying solutions). Figure 1 illustrates how these cycles were integrated into our data collection process.

### 2.1. Participants and Setting

This study was approved by the Research Ethics Committee of the Centre intégré universitaire de santé et de services sociaux de l’Est-de l’île-de-Montréal (CIUSSS-EMTL) (certificate #2025-3658). We used a purposive sampling strategy to target a diverse group of knowledge users (individuals living beyond hematological cancer, HCPs, and oncology managers) with varying characteristics (e.g., age, education) for each care pathway (HCT and CAR-T) [32]. Eligible participants living beyond hematological cancer included individuals aged 18 or older who had received a diagnosis of hematological cancer and had completed treatment with HCT or CAR-T therapy. For oncology professionals and managers, the selection criteria were: being 18 years or older and working or having worked as a HCP or manager in oncology. We identified individuals living beyond hematological cancer who were interested in participating in this project with the help of the oncology nurse navigator, hematologists from CIUSSS-EMTL, and the staff of the Cancer Partnerships Hub.

### 2.2. Data Collection

Two working groups, one for each care pathway (CAR-T and HCT), were conducted with various participants (APs, HCPs, and managers). Each group met three times (see Figure 1). The working group discussion followed the Knowledge-to-Action framework [33], focusing on the first three phases: adaptation to the local context, assessment of barriers and facilitators, and solution identification before implementation. We also refer to the definition of context as identified by Grol and Wensing [34] to guide discussions on barriers and facilitators. These include the individual care provider’s context (e.g., skills, attitudes, values, knowledge), the social context (e.g., patients’ preferences, expectations, knowledge, needs, colleagues’ attitudes and behaviors, social network culture), and the organizational context (e.g., financial resources, care organization, division of tasks). Furthermore, a patient-researcher, who had undergone two HCT procedures and works as a research assistant (LT, male), identified the key components of structured support pathways and drafted a preliminary support guide for APs who will be supporting patients with hematologic cancer undergoing allogeneic HCT. A similar process was carried out with patient partners who had undergone CAR-T cell therapy. The *Transition–Life After Cancer (Transition–La Vie Après le Cancer)* guide [35], originally designed to help patients navigate the post-treatment phase of the cancer journey, was used as a foundation for developing and adapting a structured support guide tailored to the specific needs of these two hematology care pathways (HCT and CAR-T). These draft support guides were used as the starting point for the discussions.

The first meeting began with an introduction to the research project, followed by participants completing a sociodemographic questionnaire. Subsequently, they were invited to discuss which elements should be included, adapted or removed from the support guide for patients receiving allogeneic HCT and CAR-T therapy. In the second meeting, participants shared their perspectives on the barriers and facilitators that might affect the implementation of the guide. During the third meeting, the focus shifted to identifying potential solutions and actions to facilitate the integration of the support guide into clinical practice.

The discussions from each meeting were transcribed. Alphanumeric codes were applied to the transcripts to remove any personally identifiable information and ensure participant confidentiality. The support guides from each working group were revised based on the meeting transcripts, and after each session, the updated versions were sent to participants for review on three occasions during the process. This allowed them to validate the content, provide additional context, and offer any necessary clarifications. Table 1 and Table 2 present excerpts of the structured support pathways co-designed. The complete pathways, available in French, can be accessed at: https://ihot.ciusss-estmtl.gouv.qc.ca/fr/recherche-participative-en-cours/projet-etapes-onco/etapes-onco-patients-accompagnateurs (accessed on 18 October 2025).

### 2.3. Data Analysis

We conducted an inductive thematic analysis to categorize and code the data, identifying key elements for adaptation and solutions while addressing facilitators and barriers in the CAR-T and allograft pathways [36]. One member of the research team manually coded each meeting transcript line by line. Subsequently, the team (KB, KD, LT) met to reach consensus on the coding process. We used the Supportive Care Framework [37] to guide our analysis of the anticipated needs (e.g., psychological, practical, social) highlighted during the group meetings. After coding all sessions, we grouped similar codes into potential themes and continued this process until no new themes emerged. We discussed emerging themes and preliminary findings during research team meetings, which included a patient researcher [LT] as a full member. To ensure the quality of the study, we applied criteria such as credibility (inter-rater validation between KD and KB), dependability (documentation of the research process), confirmability (transcript validation and use of excerpts), and transferability (detailed contextual description) [38].

## 3. Results

A total of 16 individuals participated in the project, including individuals living beyond hematological cancer *(n* = 9), HCPs (*n* = 6), and one oncology manager (*n* = 1).

Among the 9 individuals living beyond hematological cancer, 4 had undergone allogeneic HCT, and 5 had received CAR-T therapy. The mean age was 50 years (median: 47.5, range: 37–67) for the allogeneic HCT group and 57 years (median: 64, range: 32–71) for the CAR-T group. All 5 APs in the CAR-T group had been diagnosed with lymphoma, while in the allogeneic HCT group, 3 had leukemia and 1 had aplastic anemia. The average time since diagnosis was 13.5 years (range: 6–31 years) for the allogeneic HCT group and 5 years (range: 4–7 years) for the CAR-T group.

The 6 HCPs represented four different professions: nurse navigators (*n* = 3), hematologist (*n* = 1), a psychologist (*n* = 1), and a medical technologist (*n* = 1). The average oncology work experience was 13.5 years (range: 4–22 years). Table 3 and Table 4 report all participants’ characteristics.

Group discussions highlighted the anticipated need that APs can help address throughout the CAR-T or allogeneic HCT care pathway. They also explored the role of the AP, identified both facilitators and challenges to implement this new support model, and proposed potential solutions. The following section describes these themes in detail.

### 3.1. Needs That APs Can Help Anticipate

Discussions revealed that APs can support a broad range of needs—practical, social, emotional, informational, and psychological.

Practical concerns were often related to self-management of the illness, returning to school or work, and financial issues. One participant emphasized the importance of discussing life after treatment:

*“After the transplant, there is always something—either anti-rejection medications, maintenance chemotherapy, or other ongoing treatments. Some people experience side effects that persist over time, so this must be addressed.”*

*(P2)*

Social concerns centered around the value of being heard, sharing experiences with others, and breaking isolation through peer interaction. Participants also stressed the importance of extending support to family members and relatives. One participant noted the absence of peer connection during their own experience and welcomed the opportunity this project offers:

*“Right now, there isn’t really an option for patients to meet and get to know each other. What the patient-partner can bring is a way to fill that gap.”*

*(P3)*

Informational concerns discussed included the sharing of lived experiences and knowledge gained by APs. In the CAR-T group, participants stressed the importance of conveying realistic expectations about treatment and avoiding overly optimistic or sharing “sugar-coated” stories.

Psychological concerns were also prominent. Participants noted that APs could help patients manage fears of recurrence, feelings of lost control, and the emotional adjustment to life changes brought on by cancer. One participant shared how their life was profoundly altered upon diagnosis:

*“It was the day I got my diagnosis. The ER doctor told me, ‘Ma’am, your life has just changed from this day forward.’ That’s when I began to grieve and mentally prepare myself—because you have to come to terms with it.”*

*(P8)*

### 3.2. AP Role and Benefits

The role of the AP is primarily perceived as one of support and referral, with an emphasis on maintaining clear boundaries concerning the responsibilities of HCPs. Participants also emphasized the importance of clearly establishing personal boundaries as part of the AP role, warning that failing to do so could lead to compassion fatigue. They also noted that APs often face fluctuating health conditions—such as fatigue and immunosuppression—which require adaptive approaches to the support they provide. While many expressed a willingness to help others, some acknowledged that not all APs may feel comfortable with every aspect of the role, and that levels of competence in areas such as reassurance, coaching, and encouragement can vary. These individual differences must be recognized and clearly defined from the outset.

Furthermore, the non-professional status of APs within the clinical care team imposes inherent limitations, as reflected in the following participant’s comment:

*“Patients [receiving support] should also know that we do not claim to be doctors or nurses.”*

*(P3)*

*“We always remain within the boundaries of the patient-partner role.”*

*(P5)*

Nonetheless, participants acknowledged several potential benefits of a structured support program. These include assistance provided to both patients and APs, improved continuity and complementarity of care in relation to the work of HCPs, and a reduction in the workload of clinical staff. The guidance offered to APs, along with structured tools such as an implementation guide, was viewed as essential for ensuring effective involvement. As one HCP participant observed:

*“I see that [support from an AP] can be an added value for professionals. You know, right away, it’s an extra source of support. So personally, I see it as helpful in the long run. It will definitely lighten the workload of healthcare professionals.”*

*(P9)*

### 3.3. Challenges and Strategies for Achieving Sustainable Implementation

Challenges were discussed within the groups (Table 5) as well as their solutions (Table 6). These results were framed across four key contexts: APs, patients, HCPs, and the healthcare organization.

#### 3.3.1. AP Context

Participants noted that APs may feel pressure to help others, which could increase their risk of emotional exhaustion. To address this, they emphasized the importance of comprehensive training, external support, and practical tools. Participants also highlighted that APs should not independently identify patients to support; instead, they require guidance from the healthcare team. A further concern was the potential for APs to unintentionally share inaccurate information based on their personal experiences. To mitigate this, training and standardized tools—such as a shared lexicon—were identified as essential.

#### 3.3.2. Patient Context

In relation to patients, participants stressed the need for APs to avoid overwhelming individuals with excessive information. They emphasized the importance of providing patients with personalized, accessible information that is relevant to their needs and delivered at a pace appropriate for them.

#### 3.3.3. Healthcare Professional Context

Challenges in this context included the potential need for HCPs to reframe or clarify information provided by APs, especially to avoid misinterpretation or misinformation. Again, proper training and use of the support guide were highlighted as solutions. Participants also recommended regular reminders about each person’s role in maintaining clarity within the care team. Another challenge raised was determining the optimal timing for offering AP support to patients undergoing cellular therapy. Participants advocated for introducing AP support at multiple moments throughout the cancer care pathway.

#### 3.3.4. Organizational Context

At the organizational level, concerns were raised about ensuring the support guide remains up to date after the research project concludes. Participants suggested involving APs in quality committees within the cancer program and holding regular meetings among APs to ensure continuity and improvement of the support program.

## 4. Discussion

To our knowledge, this is the first initiative to integrate peer support (AP support) for individuals undergoing cellular therapies in Canada. Our findings underline that APs are uniquely positioned to address a wide range of unmet needs among patients who have undergone complex therapies such as CAR-T or HCT. In our study, participants, including individuals living beyond hematological cancer, HCPs, and managers, consistently emphasized the anticipated positive and tangible impact of APs’ presence. They highlighted that such support could help patients feel genuinely heard, emotionally supported, and better equipped to navigate the complexities of their care journey. This shared expectation underscores the perceived value of AP integration as a promising component of supportive care in hematological oncology context. These findings are consistent with the qualitative study by Amonoo et al. [39] which documented how patients who had undergone HCT perceived the benefits of peer support, including emotional reassurance, assistance in managing expectations and uncertainty, and broader social support. Similarly, recent studies have also shown that APs can effectively respond to emotional, informational, cognitive, and navigational needs, thereby fostering patient empowerment in the context of breast cancer care [23].

Our results underline that patients often struggle to process and retain the large volume of information they receive, confirming prior findings that patients can feel overwhelmed and unable to absorb critical medical content. In line with previous studies, our participants emphasized the need for information that is personalized, timely, and adapted to each patient’s rhythm, needs, and preferences [40,41]. As highlighted in a peer support project for individuals undergoing HCT, peers can help digest the large volume of information by translating it from their patient pocket documentation [42]. This strategy not only improves the recall and relevance of information delivered but also enhances the overall quality of care and patient experience.

However, our results also highlighted challenges related to the integration of APs—most notably, the emotional burden they experience when role boundaries are unclear, as emphasized in previous initiatives [43,44]. The solutions proposed by participants, in our study align with the findings of Chudyk et al. [45], who emphasized that the success and impact of peer support rely on clearly defined roles, appropriate training, and organizational support to prevent overstepping and compassion fatigue. In response to these challenges, our participants also recommended the use of structured tools—such as the support guide co-developed by the teams—to define roles and clarify expectations. They emphasized the importance of providing dedicated support to safeguard the well-being of both APs and patients. One example is the Cancer Partnership Hub [46], implemented in the hospital where the project took place. This initiative provides structured support to a team of APs through a combination of dedicated guidance, specialized training, and community-building activities. A resource person and a committee of practice are available to answer questions, facilitate debriefing sessions, and offer ongoing follow-up, helping APs navigate their roles effectively and remain within scope. Mandatory and ongoing training sessions focus on the AP role and the patient partnership approach, ensuring clarity around responsibilities and boundaries. Weekly meetings are held to foster a sense of community and promote resilience among APs. All APs are enrolled as volunteers (no financial compensation) and undergo background checks prior to their involvement, as well as an informal interview. Patient–AP matching is conducted by a clerk, based on similarities in diagnosis or lived experience. In cases where a match is not optimal, a reassignment process is available, coordinated by the resource person to ensure a better fit and maintain the quality of support provided. The Cancer Partnership Hub also anticipates and accommodates changes in APs’ availability. Since several APs are part of the team, flexible options allow one or more members to take a temporary or permanent pause if they experience a recurrence or need to focus on personal treatment, while maintaining continuity of the service. These measures are designed to protect APs’ well-being and ensure they are not pressured to continue beyond their capacity. Despite the innovative nature of peer support, its success relies on willingness, organization, and governance, as outlined above.

Another challenge identified in our study relates to the risk of misinformation when APs lack adequate knowledge or training, a concern echoed by Holdren et al. [47]. Consistent with the recommendations of previous studies, our findings underscore the need for APs to receive tailored training, access to evidence-based support guides and lexicons, and ongoing supervision to ensure the consistency, safety, and accuracy of the support they provide [47,48]. Once again, initiatives such as the Cancer Partnership Hub [46] could play a key role in providing high-quality training and ongoing guidance for APs alongside resources and documentation developed through the pioneering PAROLE-Onco program.

Finally, our study raised organizational concerns—particularly regarding the sustainability of the AP program beyond the research phase—which mirror the reflections found in the study by Paillard-Brunet et al. [49]. These authors highlight the importance of ethical and institutional oversight of partnership models to ensure that the contributions of APs are recognized, valued, and embedded in structured governance. In addition, Ferville et al. [50] note that HCPs’ reluctance, lack of time, and established work routines can hinder the integration of APs into clinical routine. Therefore, implementation strategies must be planned to support the long-term sustainability of the AP role. The strategies proposed in our study—such as involving APs in quality committees and holding regular meetings—offer concrete ways to overcome barriers and contribute to the long-term sustainability of such initiatives. In this regard, the implementation guide of the PAROLE-Onco initiative provides structured tools and best-practice recommendations to support the implementation, evaluation, and improvement of peer support programs, and can be adapted to other cancer trajectories [27]. This approach is fully in line with the principles of the Montreal Model of Partnership in Health [51], which emphasizes the importance of involving patients in strategic quality committees to ensure their voices are heard and to facilitate meaningful change [52].

The strength of this project lies in its co-development with both patients and HCPs. We successfully ensured the equal representation of both groups in each working session. This shared perspective enabled early negotiation of the content and boundaries of the APs’ support guides. Framing group discussions within an implementation science framework was an essential strategy to identifying potential barriers to the uptake of this innovative intervention.

However, several limitations should be acknowledged. First, one physician participated in the working group, which may influence the broader adoption of the intervention. Nonetheless, all relevant stakeholders were informed of the project and took part in informational sessions. Second, as the results are based on group discussions, quieter participants may have been less engaged or more likely to agree with the majority to avoid conflict. Lastly, these findings should be interpreted within the context of the Quebec (Canada) cancer care system, where specialized cancer services are publicly funded and accessible.

The next step in this project is to evaluate the implementation of structured support provided by APs. A multiple-case study is currently underway at a cellular therapy centre in the Montreal area. Data collection includes questionnaires, administrative data, and interviews with patients, APs, and HCPs. These results will inform the scalability and broader applicability of this promising initiative.

## 5. Conclusions

In light of our results and the existing literature, the implementation of structured support by APs—supported by structured guides and support materials—emerges as a promising response to the complex needs of individuals undergoing advanced cell therapies. This innovative initiative offers a unique opportunity to enhance the humanization of care, advance patient partnership, and promote the relational continuity that is essential to the survivorship journey of individuals living with and beyond hematological cancer.

## Figures and Tables

**Table 1:** Excerpts of the Structured Support Pathway Guide for HCT.

Keys Moments in the Care Pathway	Examples of AP Support
HCT presentation	Simplify the concept of transplantation
	Explain that transplantation is not a last-resort solution but a significant medical advancement
	Support the patient and their loved ones during the decision-making
Pre-HCT process	Demystify the relationship with the donor
	Set realistic expectations for the post-HCT period
	Advise on personal belongings to bring
HCT infusion and hospitalization	Plan times for the patient to ask questions
	Advise them to develop plans and projects for the future to cultivate hope
	Offer tips and tricks for managing symptoms (e.g., Popsicles to relieve ulcer pain)
Post HCT	Discuss the use of medication
	Encourage the patient to report symptoms and medical events
	Present GvHD in simple terms to keep the patient alert to important signs

Abbreviations: AP, Accompanying patient; HCT, Hematopoietic Cell Transplantation; GvHD, Graft-versus-host-disease.

**Table 2:** Excerpts of the Structured Support Pathway Guide for CAR-T.

Keys Moments in the Care Pathway	Examples of AP Support
CAR-T presentation and eligibility assessment	Explain the CAR-T process and pathway in simple terms
	Help gather information and ask the right questions
Pre CAR-T hospitalization	Talk about feelings of loneliness, verbalize concerns
	Talk about items to bring (e.g., calendar, book, memory games, pull-ups, vomit bags)
	Set realistic post-infusion expectations
CAR-T infusion and hospitalization	Ensure that the patient’s morale is maintained as much as possible
	Remind the patient that psychological services are always available
	If possible, visit the patient if the caregiver cannot be present, or make contact
Post CAR-T	Discuss the use of medication
	Encourage the patient to report symptoms and medical events
	Present GvHD in simple terms to keep the patient alert to important signs

Abbreviations: AP, Accompanying patient; CAR-T, Chimeric antigen receptor T-cell therapy; GvHD, Graft-versus-host-disease.

**Table 3:** Characteristics of individuals living beyond hematological cancer.

Characteristics	HCT Group (*n* = 4)	CAR-T Group (*n* = 5)
**Sociodemographic characteristics**		
**Gender**		
Male	2	1
Female	2	4
**Marital status**		
Married/cohabitation	3	4
Unmarried/divorced/widowed	1	1
**Age**		
30–40	1	1
41–50	1	0
51+	2	4
**Education level**		
High school or less	1	1
College/university	3	4
**Employment**		
Employed	3	3
Retired/student	1	2
**Clinical characteristics**		
**Type of cancer**		
Leukemia	3	5
Other (aplastic anemia)	1	0
**Time from diagnosis (years)**		
1–10	3	5
11–30	1	0
**Type of treatments received ^1^**		
Chemotherapy	4	5
Allogeneic transplant	3	5
Autologous transplant	1	2
Radiotherapy	2	2

^1^ Participants can choose more than one treatment.

**Table 4:** Characteristics of healthcare professionals and oncology manager.

Characteristics	HCT Group (*n* = 4)	CAR-T (*n* = 4)
**Sociodemographic characteristics**		
**Gender**		
Male	1	0
Female	3	4
**Age**		
30–40	3	1
41–50	1	3
**Professional experience**		
**Profession**		
Nurse navigator	1	2
Hematologist	1	0
Psychologist	0	1
Medical technologist	1	0
Oncology manager ^1^	1	1
**Years of experience**		
1–10	1	0
11–25	3	4
**Years of experience in oncology**		
1–10	2	3
11–25	2	1

^1^ One oncology manager was recruited and participated in both groups.

**Table 5:** Context-specific challenges identified.

**AP Context**	
The pressure on AP to help the person and to do well without exhausting themselves	*“APs are also patients (…). You know, sometimes there’s a strong feeling of “I need to give back.” Yes, and that’s it. It’s always about being careful not to burn myself out (…). In all cancers, not just CAR-T, they [AP] need to take care of themselves, these people. These obstacles sometimes come from wanting to give too much.” (P8*)
The initiation of contact with the patient	*“I don’t get why it [introduction] is done in a way that feels incomplete, like just saying, “I’m AP. Hello, I’m here.” They are only somewhat introduced, whereas some patients, especially those who are shyer or more introverted, might need a more proper introduction, you know?” (P9)*
The AP communicates false or incorrect information	*“A patient will ask, ‘Three months after the transplant, can I eat sushi?’ I can give them an answer, but there’s no official source where it’s clearly stated as a yes or no. (…) That’s something that’s really missing—an online resource where all the information is gathered in one place, rather than scattered across different pamphlets and printed slides.” (P4)*
**Patient Context**	
Overwhelming the patient with too much information	*Some people get stuck in their own heads. They receive a lot of information, and then they start worrying about doing things wrong” (P3)*
**Healthcare Professionals Context**	
Need to reframe different information	*“I think the fact that the patient partner and the patient have the same trajectory, the same diagnosis [can bring issues]. (…) Sometimes I must take back information that the patient has received from the other.” (P4)*
Finding the right moment to offer the structured support	*“The barriers, I would say, are the timing [the timing for introducing AP to the patient]. You know, you’re trying to find a time when the patient isn’t undergoing a thousand tests for their assessment and isn’t travelling around” (P9)*
**Organizational Context**	
Need for update of the support guide regularly	*“But that requires regular interaction with the team. It may be once a year, but between all the teams and with the patient partners.” (P4)*

**Table 6:** Suggested solutions to address challenges.

Identified Challenges	Examples of Solutions
The pressure on AP to help the person and to do well without exhausting themselves.	Creating a team of two APs to support a patient Creating a communication channel between APs Assigning tasks to determine who is most comfortable with certain situations and/or best suited based on geolocation
The initiation of contact with the patient.	Seeking assistance from the nurse navigator or a member of the medical team to initiate contact Asking the patient for their preferred method of communication and initiating contact using that method (e.g., informal email or message)
The AP communicates false or incorrect information.	Training for APs Using the developed support guide tailored to patients’ unmet needs Making the information available on an accessible webpage (hospital website) as a reference for both patients and APs Developing a standardized lexicon to align terminology between HCPs and APs, ensuring consistency in shared information
Overwhelming the patient with too much information	Breaking down information into smaller sections tailored to the patient’s needs, allowing them to choose which topics to address Developing a roadmap of the documents and educational materials the patient has received Making the information available on an accessible webpage (hospital website)
Need to reframe different information	Preparation and training Using the developed support guide tailored to patients’ unmet needs A reminder of everyone’s roles
Finding the right moment to offer the structured support	Offer it multiple times to the patient at different stages of their care journey
Need for update of the support guide regularly	Team stability Presence of AP representatives Regular meetings and frequent communications

## Data Availability

The data supporting the findings of this study are not publicly available due to privacy and ethical restrictions.

## Abbreviations

The following abbreviations are used in this manuscript:

APs	Accompanying patients
CIUSSS-EMTL	Centre intégré universitaire de santé et de services sociaux de l’Est-de l’île-de-Montréal
CAR-T	Chimeric antigen receptor T-cell therapy
GvHD	Graft-versus-host-disease
HCT	Hematopoietic Cell Transplantation
HCPs	Healthcare professionals
